# Nanofiltration Ceramic Membranes as a Feasible Two‐Pronged Approach toward Desalination and Lithium Recovery

**DOI:** 10.1002/gch2.202300151

**Published:** 2024-01-25

**Authors:** Chin Ho Kirk, Chiang Yon Douglas Chong, Xingyang Wang, Jianguo Sun, Qi Zhao, John Wang

**Affiliations:** ^1^ Department of Material Science and Engineering Faculty of Engineering National University of Singapore Singapore 117574 Singapore; ^2^ ST Engineering Advanced Material Engineering Pte. Ltd. Singapore 619523 Singapore; ^3^ National University of Singapore (Chongqing) Research Institute Chongqing Liang Jiang New Area Chongqing 401120 China

**Keywords:** ceramic membranes, desalination, lithium recovery, nanofiltrations

## Abstract

Ceramic membranes are taking center stage for separation technologies in water treatment. Among them, ceramic nanofiltration membranes are at the forefront of membrane technologies. The desalination of seawater using ceramic nanofiltration membranes is a potential application toward increasing the global water supply and tackling water scarcity. However, while the high fabrication cost poses a challenge to their large‐scale applications, high‐value separation applications can help to offset the overall cost. In this regard, ceramic nanofiltration membranes can also be explored as a viable option for high‐value lithium extraction from the waste seawater brine. In order to determine the potential of nanofiltration ceramic membranes for desalination and lithium recovery from seawater, the current efficiency of salt rejection across various operation parameters must be thoroughly evaluated. Specifically, the interactions between the Donnan exclusion, steric exclusion, zeta potential, and salt concentration play an important role in determining the salt rejection efficiency. Several strategies are then proposed to guide ceramic nanofiltration membranes toward potentially practical applications regarding desalination and lithium recovery.

## Graphical abstract

Nanofiltration ceramic membranes stand at the forefront of ceramic membrane technologies. Their deployment in desalination and lithium recovery forms a potentially high‐value application. Prior research underlined the crucial interactions between Donnan exclusion, zeta potential, and salt concentration in determining overall salt rejection. Building upon this understanding, three viable strategies are suggested: Multi‐NF, lithium recovery, and the Janus configuration.

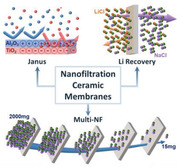

## 1 Water Scarcity, Desalination, and Lithium Recovery

Water scarcity is an urgently pressing global challenge that is being exacerbated by the ongoing effects of climate change. The ever‐increasing frequency and severity of climate‐related phenomena, notably prolonged droughts, have led to a rapid decline in both the availability and quality of water resources. As a result, one in four cities worldwide is already struggling with water insecurity.^[^ ^1^ ^]^ Moreover, the demand for water consumption is projected to rise by 50–80% over the next three decades, worsening the strain on current water resources.^[^ ^2^, ^3^ ^]^ Hence, it will be vital to expand freshwater production to meet the increasing demands of both domestic consumption and industrial use. Desalination is one of the key solutions to meet the ever‐increasing demand, with an estimated global production of 90 million m^3^ freshwater per day.^[^ ^4^ ^]^ Typically, seawater desalination accounts for the majority (≈60%) of global desalination processes, while brackish groundwater desalination accounts for ≈20%.^[^ ^5^ ^]^ Over the past two decades, thermal desalination has been gradually phased out due to the high energy demands required to evaporate seawater into vapor.^[^ ^6^ ^]^ Instead, reverse osmosis (RO) membranes are now the favored solution (≈70%) for desalination because of the significantly lower energy consumption.^[^ ^7^ ^]^

**Figure 1:**
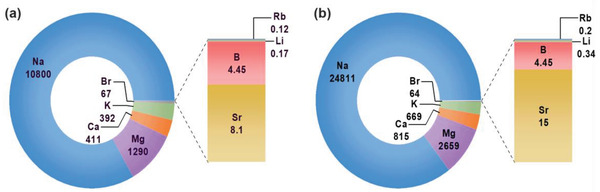
Mean concentration (mg L^−1^) of elemental species in a) seawater, b) RO brine after seawater desalination. Data extracted from ref. [9].

Desalination via RO membranes is a pressure‐driven process that separates dissolved salts from water molecules through a membrane module. Although the transition from thermal desalination to membrane desalination has provided substantial energy savings, the waste brine remains an environmental concern, especially when the brine is discharged back into the ocean.^[^ ^8^ ^]^ Instead of disposal, waste brine can be a promising source of valuable metals, such as magnesium, and lithium.^[^ ^9^ ^]^ The conventional extraction of such metals from land mining has rapidly depleted the supply of high‐grade ores, which gradually increased mining costs.^[^ ^10^ ^]^ Coupled with the low recycling rates of such metals, a recent perspective discussed the possibility of facing a supply shortage, and identified magnesium and lithium as metals that are “most critical for recovery from water”.^[^ ^11^ ^]^ Presently, there are several possible technologies that attempt to recover precious metals from seawater and brine, such as nanofiltration, electrodialysis, membrane distillation, crystallization, carbonation, electrolysis, evaporation, and adsorption.^[^ ^9^ ^]^ However, most of these technologies tend to suffer from poor profitability due to the low metal concentration even in concentrated seawater brine (**Figure** **1**).^[^ ^9^, ^11^ ^]^ Therefore, the scalability of metal recovery from desalination brine is highly dependent on the cost reduction of separation processes.

One feasible technology for desalination and metal recovery is nanofiltration (NF). Although the high salt concentration of seawater makes it harder for NF membranes to achieve complete salt rejection, NF membranes only require one‐tenth of the transmembrane pressure (TMP) during operation as compared to RO membranes.^[^ ^9^ ^]^ Furthermore, recent research has shown that NF membranes have higher water permeability, and high rejection of bivalent ions when used for desalination applications.^[^ ^12^ ^]^ For NF membranes, Donnan exclusion remains the main strategy for salt rejection. When salt dissolves in water, a membrane with a positive surface charge repels ions with the same charge (co‐ion) and attracts ions with the opposite charge (counterion). The repulsion of the co‐ion is coupled with the electroneutrality effect to bind the counterion in the feed and achieve salt rejection. This highlights the importance of the membrane surface charge in determining the effective salt rejection capability of the membrane. Additionally, the membrane surface charge is determined by the isoelectric point (IEP) of the membrane material, whereby an operating pH above the IEP would form a negative membrane charge, while an operating pH below the IEP would form a positive membrane charge. The conventional material for NF polymeric membranes is polyamide, which has a low IEP and is negatively charged at a common operating pH of 6. Consequently, the bivalent cation rejection becomes more difficult due to the electrostatic attraction to the membrane surface. As such, the NF polymeric membrane research has been focused on the development of positively charged membranes for better rejection of cations like cationic dyes and bivalent cations.^[^ ^13^ ^]^

In contrast, the IEP of common ceramic materials is higher, at 8.6–9.5 for Al_2_O_3_, and 6.1–6.6 for ZrO_2_ and TiO_2_,^[^ ^14^, ^15^, ^16^, ^17^, ^18^ ^]^ making them positively charged at pH 6. The naturally positive surface charge on ceramic membranes is one of the key motivators for the development of NF ceramic membranes. Furthermore, ceramics are acid‐resistant and can be used under mildly acidic operations to amplify the magnitude of the positive surface charge. Meanwhile, polyamide membranes are weak toward acidic solutions, and the amide linkages undergo acid‐catalyzed dissociation to permanently damage the membrane structure.^[^ ^19^ ^]^ Other tangible benefits of using ceramics over polymers in the context of nanofiltration include higher water permeability, higher mechanical strength, increased temperature resistance, the absence of pore swelling, and insensitivity toward salinity.^[^ ^4^ ^]^ Modifying the conventional polyamide membrane to overcome each of the drawbacks is arguably a fundamental challenge, and each additional layer of modification introduces a new level of complexity in the fabrication process. Therefore, the multitude of advantages of ceramics make it appealing for further development in nanofiltration applications. However, the fabrication scaling‐up of NF ceramic membranes is a challenge, due to the relatively high fabrication cost with limited membrane area. The current leading commercial NF ceramic membrane producer is Inopor (Germany), with a molecular weight cut‐off (MWCO) of 450 Da, a membrane length of 1.2 m, and an overall membrane area of 0.4298 m^2^ per membrane element. While many small‐scale pilot plants using commercial NF ceramic membranes have been built, with the largest in Canada at an overall membrane area of 234 m,^2[^ ^20^ ^]^ larger pilot plants were not considered, due to the high cost of NF ceramic membranes.^[^ ^21^ ^]^ Obviously, the high cost of building an NF ceramic plant will not be justified unless the value of the product can exceed the cost. Thus, higher‐value applications should be considered to offset the cost of such membranes. Desalination, and as an extension, metal recovery, are two potentially high‐value applications for consideration. We focus on three main metal salts – NaCl as a by‐product of desalination, followed by MgCl_2_ and LiCl as high‐value salts for metal recovery.

In this review, we have conducted a critical evaluation of progress made thus far regarding the development of NF ceramic membranes toward salt rejection. Our analysis involved consolidating the existing literature to investigate the interaction between different operational parameters affecting salt separation. Moreover, we examined the mechanisms of salt separation and quantified newer insights from the available literature. The identified shortcomings of the current technology were also thoroughly explained. We then conclude by highlighting appropriate strategies that could be implemented for both desalination and lithium recovery applications using the current state of NF ceramic membranes through a two‐pronged approach.

## 2 Current Progress and Mechanisms

**Figure 2:**
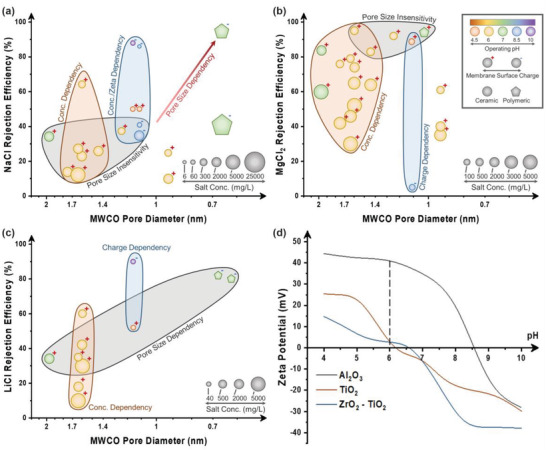
Single salt rejection efficiencies of a) NaCl, b) MgCl_2_, c) and LiCl. The experimental values are shown in Table 1. d) Zeta potential ranges for three common ceramic materials, Al_2_O_3_, TiO_2_, and ZrO_2_. Data extracted from refs. [15, 18, 22].

It is clear that the current development of NF ceramic membranes is insufficient toward desalination or metal recovery applications on large scales. Moreover, research into NF ceramic membranes remains rather scarce and tends to focus on single‐salt solutions as a measure of membrane efficiency. However, cross‐examination of the results from various literature can provide new insights and reveal possible pathways for NF ceramic membrane desalination. This is important because there are many parameters that affect the salt rejection efficiency, such as salt concentration, MWCO, solution pH, membrane surface charge, and TMP. Moreover, many studies only consider one or two variables during their experimentation. As such, a holistic overview of how each parameter interacts with one another is fundamental. **Figure** **2** shows a compilation of the available data regarding the salt rejection of NaCl, MgCl_2_, and LiCl for NF ceramic membranes. Firstly, unlike conventional figures that compare rejection efficiency to pure water permeability, a comparison between the rejection efficiency and the MWCO pore diameter is chosen instead. This is because the permeability of an NF ceramic membrane should not be as important if the salt rejection capabilities are poor. As such, it would be more crucial to examine the possible effects of pore diameter on the rejection capabilities of an NF ceramic membrane. Secondly, the effect of TMP is ignored in this figure because it has been established that an increase in TMP will only raise the salt rejection marginally, and the effect tends to reach an asymptote at 8–10 bar.^[^ ^16^, ^22^ ^]^

Due to the dependence of Donnan exclusion on the salt rejection capabilities, the salt concentration plays a major role in the rejection efficiency of an NF ceramic membrane. The most common NaCl concentration used in previous works was 2000 mg L^−1^, which is one‐fifth of the seawater concentration, and roughly one‐tenth of RO brine concentration.^[^ ^9^ ^]^ Raising the salt concentration poses growing challenges because this will increase the ionic strength of the solution, compress the electrochemical double layer, and weaken the ion rejection via Donnan exclusion.^[^ ^21^ ^]^ For example, an increase in the NaCl concentration from 300, 2000, to 5000 mg L^−1^ would reduce the NaCl rejection efficiency from 65, 35, to 12%, respectively (Figure 2a).^[^ ^16^, ^23^ ^]^ A similar concentration dependency on the salt rejection can also be observed for MgCl_2_ and LiCl (Figure 2b,c). Thus, the effective rejection of salts using NF ceramic membranes is heavily dependent on the salt concentration of the original feed. It is worth noting that at a constant NaCl concentration of 2000 mg L^−1^, the MWCO pore diameter has minimal impact on the NaCl rejection at the range between 1.1 to 2 nm, with a low rejection range of 23 to 35%. This suggests that Donnan exclusion is insensitive to the pore diameter of an NF ceramic membrane, and can be explained by the lack of ion rejection via stearic exclusion, as the Na^+^ and Cl^−^ ions have a much smaller hydrated radius of 0.358 and 0.332 nm, respectively.^[^ ^14^ ^]^ Instead, ion rejection via stearic exclusion only takes effect when the MWCO pore diameter is <0.65 nm (≈200 Da),^[^ ^24^ ^]^ achieving a high rejection of 95% at a NaCl concentration of 5000 mg L^−1^.^[^ ^25^ ^]^ Unfortunately, the actual fabrication of NF ceramic membranes with an MWCO pore diameter less than 0.65 nm poses a significant challenge to any conventional processing techniques, creating a bottleneck toward higher salt rejection efficiencies.

Another phenomenon observed was the dependence on the magnitude of the zeta potential on the salt rejection efficiency at lower NaCl concentrations. As mentioned earlier, the IEP of Al_2_O_3_ is 8.6‐9.5, while the IEP of ZrO_2_ and TiO_2_ is 6.1‐6.6. At a common operating pH of 6, the magnitude of zeta potential for Al_2_O_3_ (≈40 mV) would be significantly higher as compared to ZrO_2_ (≈3 mV) or TiO_2_ (≈3 mV) (Figure 2d).^[^ ^15^, ^16^, ^18^, ^22^ ^]^ Consequently, at the same NaCl concentration of 300 mg L^−1^, an Al_2_O_3_ NF membrane would possess a much higher salt rejection efficiency of 64% versus 25% for ZrO_2_ and 29% for TiO_2_.^[^ ^14^, ^16^, ^17^ ^]^ Similar results have been confirmed for both MgCl_2_ and LiCl, where Al_2_O_3_‐based NF membranes have higher salt rejection as compared to TiO_2_‐based NF membranes at an operating pH of 6 (Figure 2b,c). Furthermore, correlating the salt rejection toward the magnitude of zeta potential means that the polarity of the membrane surface charge is of lesser importance, especially for monovalent salts like NaCl. In another study with a NaCl concentration of 60 mg L^−1^, a TiO_2_ NF membrane showed a higher NaCl rejection at pH 10 (88%) as compared to pH 4.5 (50%), because the magnitude of the zeta potential at pH 10 (‐33 mV) was larger than pH 4.5 (+23 mV), which made the respective Cl^−^ ion repulsion stronger than the Na^+^ ion repulsion.^[^ ^22^ ^]^ As such, high NaCl rejection efficiency can also be achieved with NF ceramic membranes with a negative surface charge. However, in contrast to monovalent salts, the surface charge of the membrane becomes exceedingly important when separating multivalent cation salts, such as MgCl_2_. Due to the 2^+^ charge in Mg^2+^ ions, the Donnan exclusion effects are amplified. This results in a strong electrostatic repulsion when the membrane surface is positively charged, with a high MgCl_2_ rejection of 89%.^[^ ^21^ ^]^ Conversely, when the membrane surface is negatively charged, there will be a strong electrostatic attraction of Mg^2+^ ions toward the membrane, resulting in a low MgCl_2_ rejection of 5%. Therefore, although much literature has simply claimed that NF ceramic membranes possess better cation rejection via Donnan exclusion because they have a positive membrane surface, the statement is not completely accurate. Rather, cross‐examination of various literatures suggested that the magnitude of the zeta potential also plays a major role in controlling salt rejection, specifically toward monovalent salts.

In summary, the current state‐of‐the‐art NF ceramic membranes can have potential usage in low salinity conditions, especially for the separation of multivalent cations. Meanwhile, achieving a high rejection efficiency of monovalent salts remains a challenge, although there are certain nuances that can significantly improve salt rejection at lower salt concentrations. It is important to note that poorer salt rejection rates do not strictly mean that the technology is unfeasible. Instead, the disparity between different salts and different operating conditions presents an opportunity toward a certain level of selectivity. Further research into NF ceramic membranes should focus on the merits discussed in this section, such as the manipulation of zeta potential via pH to achieve higher salt rejection, the higher zeta potential magnitude of Al_2_O_3_ versus TiO_2_, and the flexibility of membrane pore size in the NF range (1.1 to 2 nm) to achieve similar salt rejection efficiency.

## 3 Potential Trend of Future Developments

**Figure 3:**
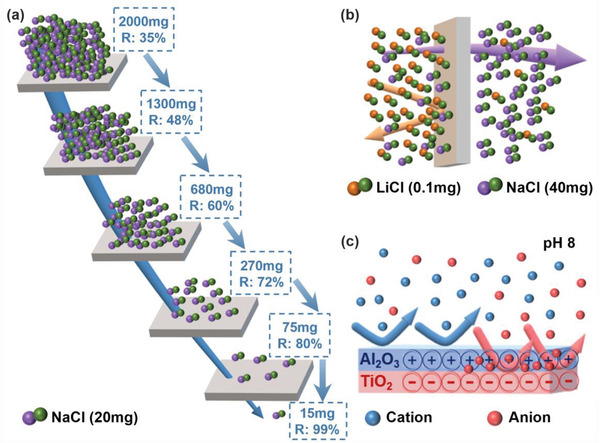
a) An illustration of a multistage NF ceramic membrane setup based on existing NaCl rejection data from Table 1 (R: Rejection). b) An illustration showing the ideal salt separation between high‐concentration NaCl and low‐concentration LiCl. c) A Janus NF ceramic membrane featuring a positively charged Al_2_O_3_ separation layer and a negatively charged TiO_2_ interlayer at pH 8.

While research into NF ceramic membranes appears to have stagnated in their development over the years, and the issue of low salt rejection seems inherent toward NF ceramic membranes, we pose a crucial question: Can the ongoing advancements in NF ceramic membranes yield practically large‐scale applications in desalination and metal recovery? Prior investigation using NF polymeric membranes has shown that two NF modules (NF‐NF) can be applied in series to achieve similar salt rejection rates as RO membranes but at reduced energy consumption. Through the optimization of operational parameters such as feed temperature, TMP, and flow rate, an NF‐NF setup was able to reduce the total dissolved solids (TDS) from ≈35 000 mg L^−1^ to ≈200 mg L^−1^, with a low energy consumption of 1.75 kWh m^−3^ (3.5 bar) for the first stage and 0.6 kWh m^−3^ (2 bar) for the second stage.^[^ ^28^ ^]^ This is a 35% reduction in total energy consumption as compared to conventional RO membranes, which requires a minimum energy consumption of 3.6 kWh m^−3^ for seawater with similar TDS concentrations.^[^ ^29^ ^]^ Other benefits of using dual‐stage NF desalination include lower energy consumption for the NF second stage due to the reduced osmotic pressure and higher operational flux. Moreover, lower capital cost and footprint are required because fewer membrane elements are needed to meet the same total flux as compared to RO.^[^ ^12^, ^29^ ^]^ However, this strategy becomes feasible because the low MWCO (<0.65 nm, 200 Da) of NF polymeric membranes allows salt rejection via a combination of Donnan exclusion and stearic exclusion mechanisms.^[^ ^30^ ^]^ Due to the larger pore diameter of NF ceramic membranes, the salt rejection only occurs via Donnan exclusion, resulting in a low maximum rejection efficiency of ≈35% at a NaCl concentration of 2000 mg L^−1^.^[^ ^21^ ^]^ Nevertheless, a multi‐stage NF ceramic membrane setup can still achieve a high overall salt rejection, based on the existing salt rejection data provided. A four‐stage NF ceramic membrane setup can reach a hypothetical rejection efficiency of 95%, while a five‐stage NF ceramic membrane setup can reach up to 99% rejection efficiency, based on the existing data (**Figure** **3**a). Given that successive NF membrane modules require less energy consumption, the concept of a multi‐stage NF ceramic membrane desalination system can be economically viable. Regardless, this only applies to low‐salinity conditions, and the actual desalination of seawater using NF ceramic membranes will still be dependent on the fabrication of ceramic membranes with much smaller pore diameters.

The metal recovery of various salts from seawater and brine is dependent on the difference in ion rejection to form a selectivity ratio. However, due to the limited number of studies conducted for mixed salt rejection using NF ceramic membranes, further discussion would be extrapolated using existing data from single salt rejection efficiencies as shown in **Table** **1**. Recently, the ion separation of Mg^2+^ and Li^+^ from salt‐lakes with a high Mg^2+^/Li^+^ ratio (MLR) >35 using NF polymeric membranes has been gaining popularity due to the rapidly growing demand for lithium‐ion batteries for electric vehicles and energy storage,^[^ ^26^ ^]^ which will definitely be continuing in the coming two decades. As expected, the initial selectivity of Li^+^ using NF polymeric membranes was poor due to the strong electrostatic attraction of the ions to the negatively charged membrane surface.^[^ ^31^ ^]^ However, after the further development of positively charged NF polymeric membranes, a high selectivity of >400 can be achieved, due to the stronger electrostatic repulsion of bivalent Mg^2+^ ions versus monovalent Li^+^ ions. Similarly, the positively charged NF ceramic membranes demonstrate higher salt rejection for MgCl_2_ as compared to LiCl. Consequently, they are also expected to perform well in the separation of Mg^2+^ and Li^+^ ions in salt‐lake brine. Even so, the problem with metal recovery in desalination brine is the significantly higher MLR, which can go up to ≈8000, due to the low LiCl concentration of ≈0.34 mg L^−1^.^[^ ^9^ ^]^ Moreover, the Na^+^/Li^+^ ratio in desalination brine is almost one magnitude higher, at 74 000, which further exacerbates the issue. Other than the massive discrepancy in the salt concentration, the separation of NaCl and LiCl monovalent salts using NF ceramic membranes presents a challenge. The rejection rates for these salts tend to have minimal differences, which is typically ≈2% regardless of the membrane's surface polarity.^[^ ^15^, ^22^, ^23^ ^]^

**Table 1:** Selected NF ceramic membrane salt rejection efficiencies. *The MWCO to pore diameter conversion is estimated based on the Stokes equation. ^†^NaCl salt rejection values referenced for Figure 3a. ^‡^LiCl salt rejection value referenced for Figure 3b.

No.	Membrane Material	MWCO Pore Diameter* [nm]	Pure Water Permeability [L m^−2^ h^−1^ b^−1^]	IEP	Operating pH	Membrane Charge	Salt	Concentration [mg L^−1^]	Rejection [%]	Pressure [bar]	Year	Reference
1	TiO_2_ (Inopor)	1.11	20	6.3	4.5 8.5 8.5 8.5 8.5 8.5 8.5 4.5 8.5	+ − − − − − − + −	NaCl NaCl NaCl NaCl NaCl NaCl NaCl MgCl_2_ MgCl_2_	60 2000 1300 680 270 75 6 100 100	50 35^†^ 48^†^ 60^†^ 72^†^ 80^†^ 86 89 5	3 3 3 3 3 3 3 3 3	2021	[21]
2	Al_2_O_3_	1.97	26.4	8.6	7.0 7.0 7.0 7.0	+ + + +	NaCl LiCl MgCl_2_ MgCl_2_	2000 2000 6000 2000	34.3 34.1 60 83.9	5 5 5 5	2018	[15]
3	TiO_2_ / ZrO_2_	1.24	0.045	6.5	6.0 6.0	+ +	NaCl MgCl_2_	300 500	37.5 92	8 8	2018	[18]
4	Al_2_O_3_	1.60	24.8	9.5	6.0 6.0 6.0 6.0 6.0 6.0 6.0 6.0 6.0 6.0	+ + + + + + + + + +	NaCl NaCl LiCl LiCl LiCl LiCl MgCl_2_ MgCl_2_ MgCl_2_ MgCl_2_	2000 300 5000 2000 500 40 5000 3000 2000 500	23 64.4 30 42 60 73 52 65 74 95.2	9 9 9 9 9 9 9 9 9 9	2015	[16]
5	TiO_2_	1.44	8	6.5	6.2 6.2 6.2 6.2	+ + + +	NaCl NaCl MgCl_2_ MgCl_2_	2000 300 3000 500	26 29 64 83	6 6 6 6	2015	[17]
6	TiO_2_ / Pd	1.64	10	6.5	6.2 6.2 6.2 6.2 6.2 6.2 6.2 6.2	+ + + + + + + +	NaCl NaCl LiCl LiCl LiCl MgCl_2_ MgCl_2_ MgCl_2_	5000 2000 5000 2000 500 5000 3000 500	12 17 10 18 35 30 46 79	6 6 6 6 6 6 6 6	2015	[23]
7	ZrO_2_	0.93 1.75	0.21 0.3	6.1 6.1	6 6 6 6 6 6 6 6	+ + + + + + + +	NaCl NaCl MgCl_2_ MgCl_2_ MgCl_2_ NaCl MgCl_2_ MgCl_2_	2000 300 3000 2000 500 2000 3000 500	10 25 35 40 61 14 42 76	8 8 8 8 8 8 8 8	2012	[14]
8	TiO_2_	1.16	20	6	4.5 10 4.5 10	+ − + −	NaCl NaCl LiCl LiCl	60 60 40 40	50 88 52 90^‡^	5 5 5 5	2002	[22]
9	PSS / PAH	0.68 0.62	6 6	4.1 5.3	7 7	− −	LiCl LiCl	500 500	82 80	4 4	2022	[26]
10	PA / TiO_2_	1.03	26.4	–	7	+	MgCl_2_	2000	94.3	4	2018	[27]
11	PA (NF90)	0.66 ^[^ ^24^ ^]^	10.16	2	7 7 7	− − −	NaCl NaCl NaCl	25 000 10 000 5000	41 73 95	9 9 9	2005	[25]

Nonetheless, we have observed a unique phenomenon with an NF Al_2_O_3_ membrane that has almost double the salt rejection with LiCl (42%) as compared to NaCl (23%), at a salt concentration of 2000 mg L^−1^.^[^ ^16^ ^]^ One reason for this effect could be the solution‐diffusion model of salt separation at lower TMP. The Li^+^ ion has a higher hydration energy of 636 kJ mol^−1^ as compared to 454 kJ mol^−1^ for Na^+^.^[^ ^32^ ^]^ This makes it harder to extract the solute Li^+^ ion from water, resulting in a higher rejection of LiCl. Furthermore, LiCl has a smaller diffusion coefficient of 1.37 × 10^−9^ m^2^ s^−1^, while NaCl has a higher diffusion coefficient of 1.63 × 10^−9^ m^2^ s^−1^.^[^ ^22^ ^]^ A higher diffusion coefficient facilitates the salt transport through the membrane, leading to higher permeation of NaCl as compared to LiCl. We also hypothesize that this effect has some interactions with the magnitude of zeta potential as discussed earlier, because the diffusion coefficient and hydration energy are constants, but the difference in rejection of LiCl and NaCl was not observed in other TiO_2_ and ZrO_2_ NF ceramic membranes. Considering that the concentration of LiCl in desalination brine is ≈0.34 mg L^−1^,^[^ ^9^ ^]^ the single salt rejection of LiCl using NF ceramic membranes is expected to be at least >90%.^[^ ^22^ ^]^ By leveraging the high concentration and low rejection of NaCl (2000 mg L^−1^, 23%), with the low concentration and high rejection of LiCl (0.34 mg L^−1^, >90%), one can enhance the difference of rejection to ≈65%. The improvement in rejection difference is thus four times higher (15%) as compared to those of commercial NF polymeric membranes,^[^ ^32^ ^]^ and provides the possibility of salt separation by selectively permeating NaCl while concentrating LiCl in the retentate (Figure 3b). Thus, NF Al_2_O_3_ membranes may be able to attain some degree of selectivity between NaCl and LiCl, thereby achieving lithium recovery. These observations greatly increase the prospects of lithium recovery using NF ceramic membranes. Given that the amount of lithium in seawater is 5000 times more as compared to land sources^[^ ^33^ ^]^ and combined with the ever‐increasing demand for lithium‐ion battery applications,^[^ ^26^ ^]^ the data provided warrants a further look into the feasibility of lithium recovery from seawater and desalination brine. Still, the actual separation of mixed salt solutions may show different results due to operational parameters, such as TMP, pH, and ionic strength interactions.

To further strengthen the prospects of desalination and metal recovery, we also propose the integration of Janus‐type membranes into NF ceramic membranes. In the context of nanofiltration, Janus membranes have an oppositely charged interlayer and separation layer.^[^ ^34^ ^]^ This distinctive feature allows the simultaneous electrostatic repulsion of both cations and anions, leading to a highly effective salt rejection, although the exact rejection mechanism requires further investigation.^[^ ^35^ ^]^ Based on prior studies with Janus NF polymeric membranes, the asymmetrically charged membrane layers were able to achieve a high rejection (>95%) for both bivalent cations (MgCl_2_, CaCl_2_) and bivalent anions (Na_2_SO_4_, MgSO_4_).^[^ ^36^ ^]^ A recent review suggested that the enhancement in multivalent ion rejection stemmed from the retention of the counterion within the intermediate layer of the membrane. For example, a negatively charged membrane would repel anions, and attract cations, causing the cation to permeate through the top layer. The positively charged secondary layer will then repel the cation, leading to an accumulation of cations within the membrane layers, and further improving the overall salt rejection.^[^ ^34^ ^]^ This gradual accumulation of counterions in the intermediate layer also increases the rejection of monovalent salts. Notably, the NaCl rejection of Janus NF polymeric membranes can increase from 47%, 52%, to 54% after filtration durations of 10, 60, and 120 s respectively.^[^ ^37^ ^]^

Conversely, research into Janus ceramic membranes has been fairly limited so far, and most of the existing literature focused on the fabrication of asymmetric wettability membranes (hydrophobic/hydrophilic) for oil‐water separation, instead of asymmetric charge membranes (positive/negative) for ion rejection. Indeed, we would like to emphasize that the methods to fabricate Janus NF ceramic membranes are already established, even though they have not been explicitly referred to as such in the current literature. For example, based on the zeta potential of TiO_2_ and Al_2_O_3_ within the pH range of 7 to 9, TiO_2_ will exhibit a negative charge, while Al_2_O_3_ will exhibit a positive charge (Figure 2d). This would imply that a positive‐negative Janus NF ceramic membrane can be fabricated using an Al_2_O_3_ separation layer with a TiO_2_ interlayer, while a negative‐positive membrane can be achieved using the opposite configuration (Figure 3c). However, it would be crucial to exercise caution during the fabrication process to ensure that the positive‐negative interface is close enough to achieve the dual‐repulsion effect. Previous works on Janus NF polymeric membranes have demonstrated the successful fabrication featured ultrathin membranes with a separation layer thickness of 50–100 nm.^[^ ^36^, ^37^ ^]^ This would mean that the ceramic NF separation layer, typically achieved through a polymeric sol‐gel process, would need to be coated onto an opposing interlayer material. As such, the calcination of the Janus NF ceramic membrane can face challenges related to the interface mismatch. Regardless, the application of Janus NF ceramic membranes in desalination and metal recovery holds great potential to provide greater flexibility with the operational parameters. The substantial zeta potential magnitude (±30 mV) of both Al_2_O_3_ and TiO_2_ in close proximity could introduce unexpected effects with the Donnan exclusion mechanism. Such studies could contribute valuable new insights and potentially enhance the overall understanding of desalination and metal recovery using NF ceramic membranes.

In conclusion, the realization of large‐scale desalination and lithium recovery using NF ceramic membranes still lies far ahead. As a first step, further research into the fabrication and capabilities of NF ceramic membranes is crucial, because the inherent superior mechanical and chemical properties of ceramic membranes offer a direct advancement in comparison to polymeric counterparts. In addition, NF ceramic membranes also require less TMP during operation and have much higher flux as compared to RO membranes, which can effectively reduce the overall operational cost. Nonetheless, the successful implementation of NF ceramic membranes in desalination will greatly increase global water production and alleviate water scarcity challenges. Additionally, the application of NF ceramic membranes for lithium recovery from waste desalination brine offers a promising avenue to address the issue of brine disposal into oceans.

Herein, the review discusses three potential research trends that can push NF ceramic membranes toward a two‐pronged approach for desalination and lithium recovery. First, the use of a multistage NF setup can achieve an overall high salt rejection efficiency in low‐salinity (<2000 ppm) conditions to serve as a foundation for large‐scale desalination applications. This is predicated on the premise that each subsequent NF ceramic membrane deployment would demand reduced operating pressure, while also aligning with the general tendency of NF membranes to require lower operational pressures in comparison to RO membranes. Second, the data available from previous literature has shown in‐principle evidence of lithium recovery from desalination brine. By controlling the concentration of LiCl and NaCl, it is possible to extend the difference in rejection to 65%, thereby achieving effective lithium recovery. Third, the emergence of Janus ceramic membranes represents a prospective new trend that could show interesting results with the Donnan exclusion mechanism for both desalination and lithium recovery. This is due to the close proximity of the positively and negatively charged membrane layers. Ideally, both the positive and negative ion rejection will be enhanced via the large zeta potential magnitude of both Al_2_O_3_ and TiO_2_, as demonstrated by prior studies with NF polymeric membranes. Conducting further experiments and studies to validate the various hypotheses discussed in this review could prove invaluable in bridging the wide technological gaps toward the adoption of NF ceramic membranes as a two‐pronged approach for desalination and lithium recovery.

## Conflict of Interest

The authors declare no conflict of interest.
